# Analysis of Motor Neurons Differentiated from Human Induced Pluripotent Stem Cells for the Use in Cell-Based Botulinum Neurotoxin Activity Assays

**DOI:** 10.3390/toxins12050276

**Published:** 2020-04-25

**Authors:** Maren Schenke, Brit-Maren Schjeide, Gerhard P. Püschel, Bettina Seeger

**Affiliations:** 1 Institute for Food Toxicology, Department of Food Toxicology and Replacement/Complementary Methods to Animal Testing, University of Veterinary Medicine, 30173 Hannover, Germany; maren.schenke@tiho-hannover.de; 2 Institute of Nutritional Science, Department of Nutritional Biochemistry, University of Potsdam, 14558 Nuthetal, Germany; schjeide@uni-potsdam.de (B.-M.S.); gpuesche@uni-potsdam.de (G.P.P.)

**Keywords:** Botulinum neurotoxin, motor neurons, cell-based in vitro assay, potency assessment, induced pluripotent stem cells

## Abstract

Botulinum neurotoxins (BoNTs) are potent neurotoxins produced by bacteria, which inhibit neurotransmitter release, specifically in their physiological target known as motor neurons (MNs). For the potency assessment of BoNTs produced for treatment in traditional and aesthetic medicine, the mouse lethality assay is still used by the majority of manufacturers, which is ethically questionable in terms of the 3Rs principle. In this study, MNs were differentiated from human induced pluripotent stem cells based on three published protocols. The resulting cell populations were analyzed for their MN yield and their suitability for the potency assessment of BoNTs. MNs produce specific gangliosides and synaptic proteins, which are bound by BoNTs in order to be taken up by receptor-mediated endocytosis, which is followed by cleavage of specific soluble N-ethylmaleimide-sensitive-factor attachment receptor (SNARE) proteins required for neurotransmitter release. The presence of receptors and substrates for all BoNT serotypes was demonstrated in MNs generated in vitro. In particular, the MN differentiation protocol based on Du et al. yielded high numbers of MNs in a short amount of time with high expression of BoNT receptors and targets. The resulting cells are more sensitive to BoNT/A1 than the commonly used neuroblastoma cell line SiMa. MNs are, therefore, an ideal tool for being combined with already established detection methods.

## 1. Introduction

Botulinum neurotoxins (BoNTs) are a group of bacterial exotoxins, which are the most potent toxins known to occur naturally. There are seven well-studied serotypes in addition to mosaic forms and numerous subtypes, which are produced by bacteria of the genus *Clostridium* [1]. All serotypes are produced from a single polypeptide precursor cleaved into a small and a large subunit. These serotypes have very similar pathways by which they bind and enter their physiological target, peripheral cholinergic neurons, and are, ultimately, accumulated at cholinergic nerve endings [2]. The inhibition of neurotransmitter release in motor neurons (MNs) leads to a flaccid paralysis and may result in respiratory failure [3]. Among these serotypes, BoNT/A, B, E, and F can cause human botulism, either by oral ingestion of the bacterium or the toxin itself or by wound infection [4,5,6]. Low concentrations of BoNT/A1 and B1 are used to treat strabismus, spasticity, certain neurological conditions, pain disorders, or urological conditions, but it is estimated that half of the production of BoNT/A1 is used in aesthetic medicine to remove frown lines and wrinkles [7].

BoNTs have an extraordinary specificity for the neuromuscular junction, which can be explained by the dual receptor binding model described by Montecucco [8]. Initially, BoNTs adhere to gangliosides concentrated on neuronal membranes, which is followed by binding to neuronal protein receptors and subsequent endocytosis. Vesicular acidification causes the small subunit, a zinc-dependent endoprotease, to exit the endosome, detach from the large subunit, and cleave soluble N-ethylmaleimide-sensitive-factor attachment receptor (SNARE) proteins, which are essential for exocytosis. The exact cleavage site and target protein depend on the BoNT serotype [7]. A list of the most common BoNT serotypes and mosaic forms with the corresponding receptors and substrates relevant for inhibition of neurotransmission is given in Table 1.

BoNT/A and BoNT/B are produced from cultures of *Clostridia* for pharmaceutical application with batch-to-batch potency variability [14]. Combined with the severe neurotoxicity of BoNTs, this necessitates reliable methods for potency estimation. The gold standard for potency estimation is the mouse lethality assay in which mice are injected with multiple dilutions of BoNTs and the LD_50_ is determined [15]. The mouse lethality assay has been criticized for being time-consuming, expensive, not always representative for humans, and having an intra-laboratory error of up to 20% and inter-laboratory error of more than 50% [16,17]. Injection with BoNTs causes severe distress in the test animals, which should be reduced according to the 3Rs principle (Reduction, Refinement, Replacement) described by Russel and Burch [18,19]. Although in vitro methods for the potency estimation have been developed and successfully validated, the number of animals used in the mouse lethality assay has not decreased. About 400,000 mice are still used in Europe annually [20]. The prerequisite for an in vitro assay that can reduce or replace the use of the mouse lethality assay is the ability to detect functionally active toxin and to consider the processes of toxin binding, internalization, release from neuronal vesicles, and target cleavage [4,17,21]. Cell-based assays utilizing neuronal cell lines, primary cultured neurons, or stem cell-derived neurons are capable of recapitulating several if not all of these aspects [22,23]. Human MNs, as demonstrated by Pellett et al. [24], are significantly more sensitive than other types of neurons, reaching estimated IC_50_ values of 0.006 mouse LD_50_ U per well for SNARE-cleavage of BoNT/A1 and F1 [22]. All BoNT serotypes have the same very well-defined mode of action, which results in the cleavage of a SNARE protein at a cleavage site characteristic for each serotype [7]. The quantification of a specific cleaved target is incorporated in many in vitro assays, which limits their applicability to single BoNT serotypes only [17]. Cell-based in vitro assays have been validated by BoNT producing companies for the analysis of BoNT/A1 and /B1 [20]. It is expected that new BoNT serotypes will enter the market, which would require new in vitro assays [25]. To detect all serotypes in one assay, the quantification of the neurotransmitter release inhibition, which is the toxicological endpoint at the cellular level for all BoNT serotypes, has been proposed in several neuronal cell line-based assays [26,27,28]. However, for basic research, mechanistic studies, and the development of BoNT inhibitors and antibodies, human MNs would be the most physiologically relevant cell type instead of cell lines and can recapitulate every step of BoNT intoxication including neurotransmitter release inhibition [17,23,29,30,31]. Another factor to consider in terms of sensitivity is the interspecies variation. In the case of BoNT/B, the affinity to human and chimpanzee receptor synaptotagmin 2 (SYT2) is decreased due to a mutation that leads to a 10-fold higher potency of BoNT/B in mice compared to humans. This emphasizes the role of the protein receptor in regulating BoNT sensitivity and the need for models relevant to the human [13,32].

Since the reprogramming of differentiated somatic cells by viral integration of the four transcription factors OCT4, SOX2, KLF4, and c-MYC into induced pluripotent stem cells (iPSCs) was developed by Takahashi and Yamanaka [33], the methods to differentiate human MNs from stem cells have greatly advanced. With the use of small molecules that mimic endogenous signaling cues, neurogenesis can be reproduced in vitro [34]. Neuralization can be induced through dual SMAD inhibition by adding inhibitors of bone morphogenic protein (BMP) and transforming growth factor beta (TGFβ) signaling pathway, like SB431542 or Dorsomorphin, respectively, which block differentiation into mesodermal and endodermal lineages [35]. In vivo, concentration gradients of signaling molecules lead to patterning of the neural tube along the rostro-caudal and the dorso-ventral axis. If neuroectodermal cells are to be differentiated to MNs in vitro, the addition of a combination of specific concentrations of retinoic acid (RA) and a sonic hedgehog analog e.g., Purmorphamine (PMA), results in differentiation into oligodendrocyte transcription factor 2 (OLIG2) positive motor neuron progenitor cells (pMNs) [36]. After about four weeks in total, with the addition of neurotrophic factors and the aid of notch inhibitors to induce the cell cycle exit and accelerate maturation, neuronal cultures with MN yields up to 95% were reported [37].

The aim of this study was to identify differentiation protocols that can efficiently generate MNs suitable for BoNT potency estimation with cell-based assays. Differentiation protocols based on the publications by Du et al. [38], Maury et al. [39], and Kroehne et al. [40] were analyzed regarding their MN yield and their capacity to generate corresponding receptors and substrates for all BoNT serotypes compared to the neuroblastoma cell line SiMa and to the human brain. All tested protocols generated MNs, which expressed receptors and substrates for all BoNT serotypes. The protocol based on Du et al. [38] showed the highest yield of MNs. The differentiated cells were shown to be more sensitive to BoNT/A1 than SiMa cells in a Western blot analysis of cleaved Synaptosomal-associated protein 25 (SNAP25), the substrate of BoNT/A. This indicates that human MNs can be the foundation of sensitive and physiologically relevant in vitro assays for the potency estimation of botulinum neurotoxins.

## 2. Results

### 2.1. Differentiation Protocol by Du el al. for Differentiation of iPSCs to MNs Has the Highest MN Yield

**Figure 1:**
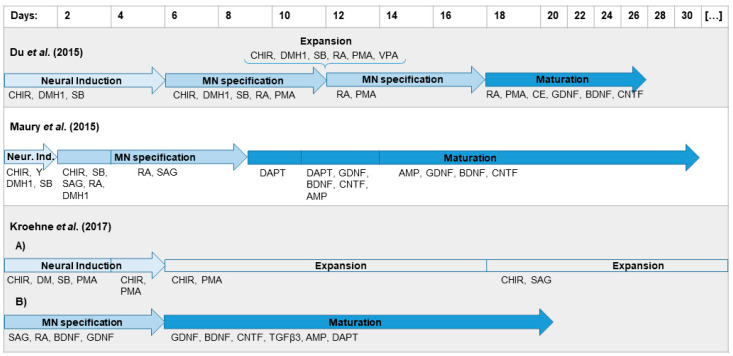
Differentiation protocols for the generation of motor neurons (MNs) from human induced pluripotent stem cells (iPSCs) as used in this study. The protocols by Du et al. [38], Maury et al. [39], and Kroehne et al. [40] were adopted with minor changes. Motor neuron progenitors (pMNs) generated on day 12 with the protocol based on Du et al. [38] can be expanded in a valproic acid (VPA) containing expansion medium for a limited time. The protocol based on Kroehne et al. [40] is divided into the (**A**) generation and expansion of a pure population of neural progenitor cells (NPCs) and (**B**) differentiation to MNs. Abbreviations for the supplements can be found in Table A1.

MNs are the physiological target cells of BoNTs and can be generated in vitro from human iPSCs as a relevant model to study the effect of BoNTs. Three differentiation protocols based on previous publications by Du et al. [38], Maury et al. [39], and Kroehne et al. [40] were compared for their capacity to generate MNs. The iPSC line IMR90 was differentiated as summarized in Figure 1.

**Figure 2:**
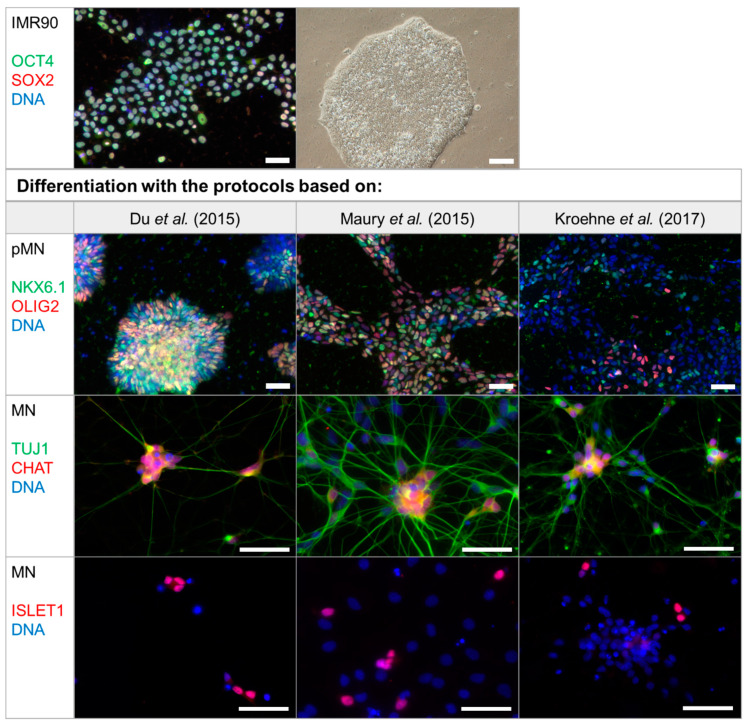
Generation of MNs from iPSC line IMR90 with protocols based on Du et al. [38], Maury et al. [39], and Kroehne et al. [40]. Representative images of the differentiation progress are shown with staining of molecular markers characteristic for the differentiation stages of undifferentiated iPSCs IMR90 (top panel), pMNs, and mature MNs (bottom panel). Undifferentiated pluripotent stem cells can be identified by nuclear expression of transcription factors SRY (Sex determining region Y) box 2 (SOX2) and octamer-binding protein 4 (OCT4) and form round colonies with smooth edges. pMNs exhibit transcription factors oligodendrocyte transcription factor 2 (OLIG2) and NK6 homeobox 1 (NKX6.1) staining in the nucleus on day 12 for the protocol based on Du et al. [38], day 9 for the protocol based on Maury et al. [39], and day 6 of MN specification for the protocol based on Kroehne et al. [40]. Mature MNs express choline O-acetyltransferase (CHAT) and insulin gene enhancer protein ISL-1 and were analyzed on day 28 of the protocol based on Du et al. [38], day 32 of the protocol based on Maury et al. [39], and day 21 of the protocol based on Kroehne et al. [40]. The pan-neuronal marker β3-tubulin (TUJ1) was used for staining of neurons. Controls for the antibodies can be seen in Figure A1. Scale bar = 50 µm.

Characteristic molecular markers were analyzed with immunocytochemistry at the stages of pluripotent stem cells, pMNs, and mature MNs (Figure 2). Pluripotency of IMR90 was shown by detection of transcription factors SRY (Sex determining region Y) box 2 (SOX2) and octamer-binding protein 4 (OCT4) in the nucleus, which are two of the factors that have been used for reprogramming into iPSCs [41]. pMNs were generated through dual SMAD inhibition followed by dorsalization and caudalization and can be identified by detection of the transcription factors OLIG2 and NK6 homeobox 1 (NKX6.1) in the nucleus (Figure 2). While most cells differentiated with the protocols based on Du et al. [38] and Maury et al. [39] are stained with at least one of these pMN markers, most cells generated with the protocol based on Kroehne et al. [40] are OLIG2 and NKX6.1 negative (Figure 2). Differentiated neurons express pan-neuronal marker β3-tubulin (TUJ1) and form extensive networks. After the cell cycle exit, the transcription factor called insulin gene enhancer protein ISL-1 (ISLET1) is expressed by MNs, which have the capability to synthesize acetylcholine after maturation. Choline O-acetyltransferase (CHAT) is required for acetylcholine synthesis, which could be detected for every protocol used.

**Figure 3:**
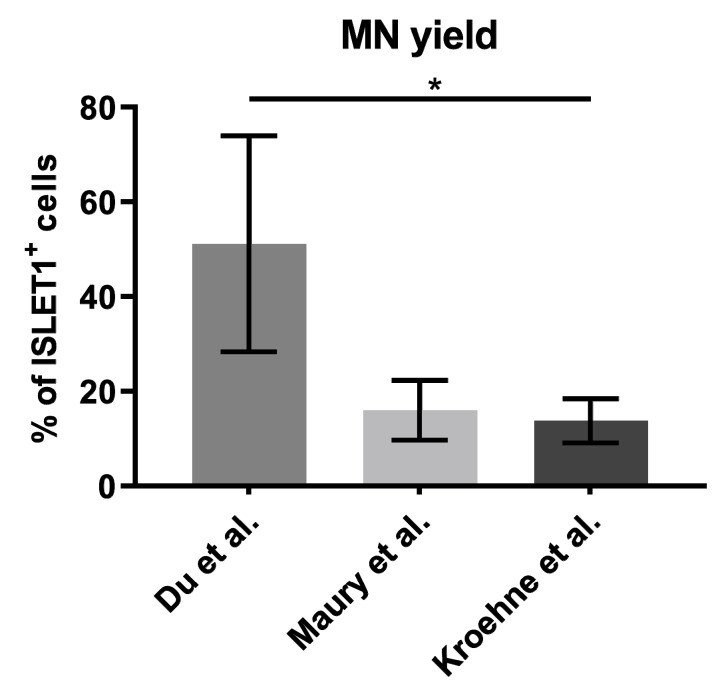
MN yield was analyzed on day 28 of the protocol based on Du et al. [38], day 32 of the protocol based on Maury et al. [39], and on day 21 of the protocol based on Kroehne et al. [40]. ISLET1-positive MNs were counted by a person blind to the experiment from an average of 200–400 cells in random fields from at least three independent differentiations. Depicted is the mean ± SD. Significant differences were found with Kruskal-Wallis test and Dunn’s multiple comparisons test (* *p* ≤ 0.05).

MNs were quantified using the nuclear marker ISLET1 (Figure 3).

The average MN yield was highest, reaching 51% (34%–84%) when the protocol based on Du et al. [38] was applied, while the protocols based on Maury et al. [39] and Kroehne et al. [40] yielded 16% (9%–21%) and 14% (9%–17%) of MNs, respectively.

### 2.2. MNs Differentiated In Vitro Exhibit the Full Panel of BoNT Serotype-Specific Substrates and Receptors

**Figure 4:**
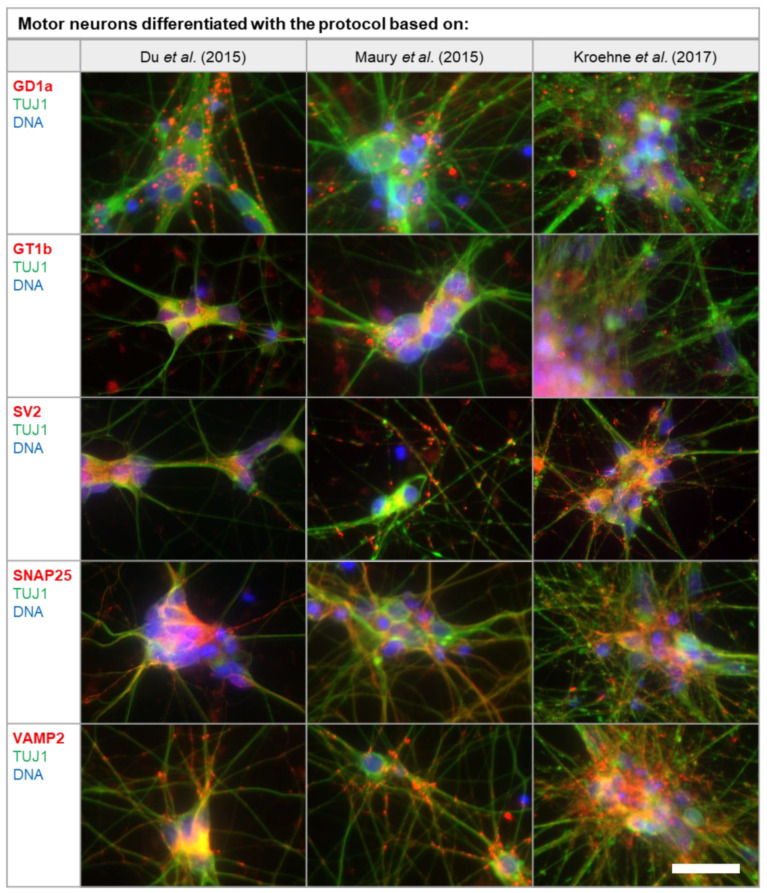
Immunocytochemical detection of BoNT targets and receptors in differentiated MNs. The ganglioside receptors GT1b and GD1a, the protein receptor SV2, as well as the substrates SNAP25 and VAMP2 were analyzed and co-stained with pan-neuronal marker TUJ1. Synaptic vesicle proteins SV2 and VAMP2 as well as gangliosides GD1a and GT1b and t-SNARE SNAP25 are concentrated at the synapses in the form of spots. Staining was conducted on day 28 of the protocol based on Du et al. [38], day 32 of the protocol based on Maury et al. [39], and on day 21 of the protocol based on Kroehne et al. [40]. Pseudo-colors were used for the staining of GD1a, GT1b, and SV2 with TUJ1, for uniform staining of TUJ1 in green and the antigen of interest in red. Controls for the antibodies can be seen in Figure A1. Scale bar = 20 µm.

Gangliosides and synaptic proteins, which are found in high concentrations at the neuromuscular end plate, are used by BoNTs in order to bind and enter neurons via endocytosis. These receptors, as well as the SNARE proteins, which are the substrate of the proteolytic subunit, are necessary for a cell to be sensitive to BoNTs. Therefore, MNs were generated in vitro and analyzed with immunocytochemistry for the presence of gangliosides GT1b and GD1a, protein receptor synaptic vesicle protein (SV2), substrates SNAP25, and the Vesicle-associated Membrane Protein (VAMP2) (Figure 4). Staining of BoNT targets GD1a, GT1b, SV2, SNAP25, and VAMP2 could be detected in the soma of the neurons as well as on the processes co-stained with pan-neuronal marker TUJ1. Although the intensity of the staining was not homogenous and not all cells were positive for the respective antigen, all protocols generated neurons that expressed these markers. For GD1a, GT1b, SV2, and VAMP2, staining can be seen in the form of spots due to the concentration in synapses. The t-SNARE SNAP25 is slightly more evenly distributed on the neuronal membrane.

**Figure 5:**
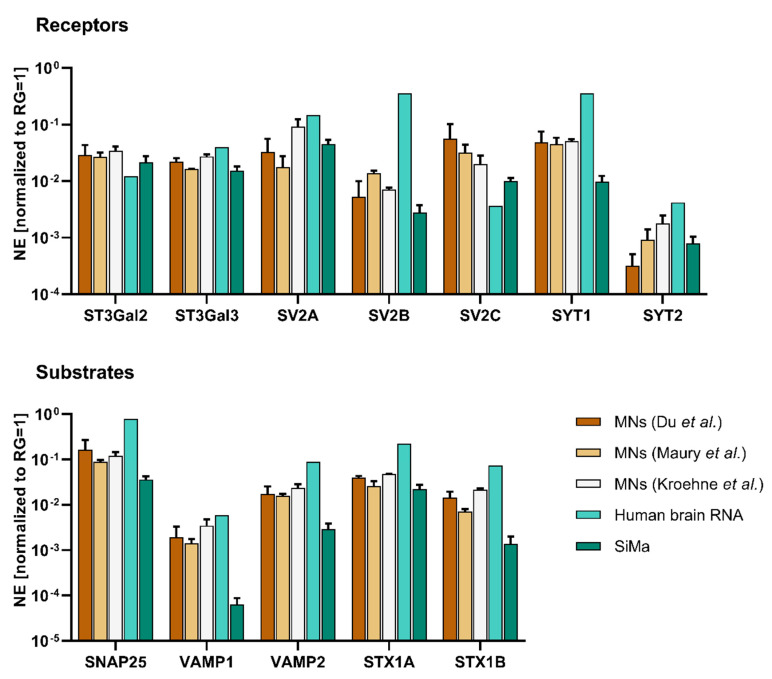
Gene expression levels of BoNT receptors and targets in MNs differentiated with protocols based on Du et al. [38], Maury et al. [39], and Kroehne et al. [40] were compared to total human brain RNA and partially differentiated SiMa cells. Gene expression levels of the GD1a and GT1b synthesizing enzymes *ST3GAL2* and *ST3GAL3* were analyzed for estimation of ganglioside expression levels. Furthermore, the protein receptors *SV2A/B/C* and *SYT1/2* as well as the receptors *SNAP25*, *VAMP1/2,* and *STX1A/B* were analyzed. Reference genes (RGs) ribosomal protein S23 (*RPS23*) and cyclophilin A (*PPIA*) were used for normalization of expression (NE). Gene expression levels in SiMa and MNs were analyzed in at least three independent differentiations. Total human brain RNA was analyzed once. Values are depicted as mean ± SD.

The expression of substrates and receptors for all BoNT serotypes (Table 1) was additionally quantified with RT-qPCR. Since the ganglioside receptors are not directly encoded by a single gene, the expression levels of the enzymes required for synthesis of GD1a and GT1b, namely ST3 beta-galactoside alpha-2,3-sialyltransferases 2 and 3 (*ST3GAL2* and *ST3GAL3*), were quantified. Expression of receptors (*SNAP25, VAMP1/2*) and targets (*SV2A-C, SYT1/2*) could be detected in the neuronal populations generated by the differentiation protocols used in this study. The gene expression levels were compared to total human brain RNA and to the partially differentiated neuroblastoma cell line SiMa, which is commonly used in cell-based in vitro assays for the potency estimation of BoNTs (Figure 5).

Nearly all genes were expressed at higher levels in total human brain RNA compared to MNs. SiMa cells often had the lowest gene expression levels. Among the MNs generated with the three protocols, mostly small differences were found. The only gene that was expressed at considerably lower levels in human brain RNA compared to MNs was *SV2C*, which is the high-affinity receptor isoform for BoNT/A [42].

### 2.3. Potency Estimation of BoNT/A with MNs Differentiated In Vitro and SiMa Cells

**Figure 6:**
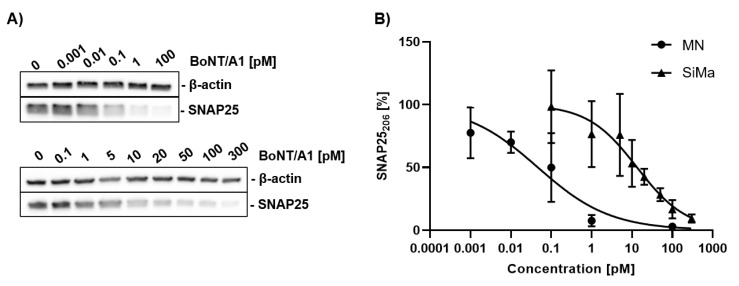
Western blot analysis of SNAP25 cleavage by BoNT/A1. (**A**) MNs (upper panel) generated with the protocol based on Du et al. [38] and differentiated SiMa cells (lower panel) were treated with different concentrations of BoNT/A1 for 48 h and analyzed via Western blot. (**B**) The proportion of uncleaved SNAP25_206_ was quantified, normalized against β-actin, and modelled by nonlinear regression (four parameters, variable slope).

A common method for evaluating BoNT activity is to treat cells with different concentrations of BoNTs and quantify the proportion of cleaved substrate via Western blot (Figure 6).

**Figure A2:**
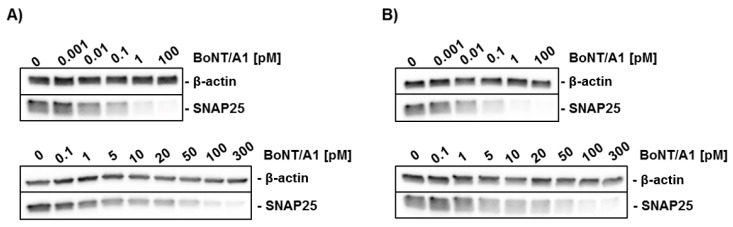
Replicates of Western blot analysis of SNAP25 cleavage by BoNT/A1 that were not shown in Figure 6. MNs (upper panel) generated with the protocol based on Du et al. [39] and differentiated SiMa cells (lower panel) were treated with different concentrations of BoNT/A1 for 48 h and analyzed via Western blot. (**A**,**B**) each depict one replicate.

MNs generated with the protocol based on Du et al. [38] and differentiated SiMa cells were treated for 48 h with BoNT/A1 and the proportion of cleaved substrate was quantified by Western blot. A SNAP25-antibody that binds only to the full-length protein SNAP25_206_ was used. Replicates that are not depicted in Figure 6 can be found in Figure A2. IC_50_-values for SNAP25 cleavage of 0.046 pM (95% confidence interval 0.0165–0.123 pM) for MNs and 13.31 pM for SiMa (95% confidence interval 7.319–22.71 pM) were detected. This translates into LD_50_-values of 0.97 and 279 mouse lethality doses per mL (MLD/mL), respectively.

## 3. Discussion

### 3.1. Differentiation Protocol Based on Du el al. for Differentiation of iPSCs to MNs Has the Highest MN Yield

Cholinergic neurons are the physiological target of BoNTs and, like many other cell types, can be generated in vitro using pluripotent stem cells. Especially through the use of human iPSCs, models relevant to humans have become easily accessible. In the case of BoNTs, human MNs generated from pluripotent stem cells are a valuable and sensitive model due to the mode of action of the neurotoxin. Once the toxin has been taken up by the organism, regardless of the route, BoNTs specifically target peripheral cholinergic nerve endings, are endocytosed and inhibit the release of neurotransmitters.

Since the differences between the BoNT serotypes (Table 1) are also conveyed by the specificity of the receptors bound and targets cleaved, and since MNs generated in vitro express these (Figure 5), MNs seem to be a good basis for research, e.g., in the field of BoNT inhibitor and antibody development. As the expression of receptors and substrates of the different BoNT serotypes varies from species to species, the use of human cells for BoNT potency testing of pharmacological products is advantageous. From this point of view, human cells are a good model for the effect on the organism, as they do not require inter-species extrapolation and test animals [30]. Human MNs from a commercial source have been shown to be very sensitive for BoNTs by Pellett et al. [24], but are rather costly.

In this study, available protocols for differentiating MNs in vitro were analyzed for their capacity to generate cells sensitive to all major BoNT serotypes. The differentiation protocols based on Du et al. [38], Maury et al. [39], and Kroehne et al. [40] all generate MNs within several weeks, however the protocol based on Du et al. [38] shows the highest yield of MNs quantified by staining the MN marker ISLET1 (Figure 2).

The yield of MNs reported in the respective publications could only be compared to a limited extent since different markers were selected for quantification. For reasons of comparability, we decided to quantify cells expressing ISLET1, which is a transcription factor found in all MNs [43]. As located in the cell nucleus, it can be reliably quantified with immunocytochemistry. For the protocol based on Kroehne et al. [40], only few pMNs were found in this study, which indicates that the transition to pMNs is incomplete. While no MN yield was reported by Kroehne et al. [40], the publication by Reinhardt et al. [44], upon which the differentiation described by Kroehne et al. [40] is based, reported 50% of motor neuron and pancreas homeobox 1 (MNX1) and TUJ1-positive cells. In comparison, only 14% ISLET1-positive MNs could be obtained on day 21 of MN differentiation in this study. The protocol based on Maury et al. [39] showed a high number of pMNs, but continued proliferation of progenitor cells might be an issue, which results in a limited proportion of MNs after maturation. This reached 16% on day 32 of the differentiation protocol. This could be overcome by the addition of NOTCH inhibitors throughout the maturation process, as was done by Du et al. [38] and in the protocol based on Kroehne et al. [40]. Unfortunately, Maury et al. [39] only quantified the yield of immature ISLET1- and MNX1-positive MNs on day 14 and report 74% yield, but did not quantify for maturated MNs. For the protocol based on Du et al. [38], a high proportion of pMNs and MNs could be detected. Du et al. [38] quantified CHAT and microtubule associated protein 2 (MAP2) expressing neurons on day 28 and reported 91% MN yield. With the protocol based on Du et al. [38] used in this study, 51% MN yield could be achieved on day 28, which is closer to the yield in the original publication than in the other protocols replicated in this study. Nevertheless, due to variations in protein expression levels and differing sensitivity of neuronal and MN markers as well as differences in the quantification methods, these yields only serve as an estimate. Each of the protocols used in this study has advantages and disadvantages in terms of yield, variability, need for manual procedures, or time required for differentiation. The protocol based on Du et al. [38] required the most manual steps during the differentiation, which might explain the high variability, but has the highest yield of MNs in our study.

### 3.2. Sensitivity for BoNTs

Two factors contribute to the specificity of BoNTs for MNs. Due to their size, BoNTs are unable to cross the blood brain-barrier, which limits their distribution mostly to peripheral neurons [45]. BoNTs could enter neurons of the central nervous system by retrograde transport. Nevertheless, high-affinity gangliosides and receptor isoforms are concentrated on MNs and the neuromuscular junction [42,46]. Different BoNT serotypes have the same mode of action, but differ with regard to the specific receptors and substrates [47]. In this study, gene expression levels of currently identified protein receptors and substrates for all BoNT serotypes were analyzed in MNs generated from human iPSCs in vitro (Figure 5). In addition, protein expression of most receptors and substrates was analyzed with immunocytochemistry. The staining seen in Figure 4 reflects the subcellular localization of the gangliosides GT1b and GD1a as well as synaptic vesicle proteins VAMP2 and SV2, which are concentrated at the synapse [46,48,49,50]. Staining of SNAP25, located at the cell membrane, is distributed more evenly along the neurons [51,52]. Not all neurons generated by one differentiation protocol are stained equally, which indicates a diverse cell population. RT-qPCR was used to quantify the respective gene expression levels. MNs generated in vitro express all receptors and substrates required for intoxication with different BoNT serotypes. In order to estimate the sensitivity to BoNTs, which depends on the expression of these receptors and substrates, we compared the gene expression levels of human MNs with human brain RNA and the neuroblastoma cell line SiMa. Human brain RNA serves as a control in this study. In general, expression levels of MNs appear to be between the levels found for human brain RNA and SiMas, which may indicate a different extent of neuronal differentiation and maturation. However, due to the higher number of synapses in the brain in combination with the fact that only half of the cells in the brain are neurons, direct comparison of expression levels found in the brain with MNs generated in vitro is difficult [53,54,55]. In addition, no pure MN populations could be generated. Persistent progenitor cells and other unidentified neuronal cell types may obscure the receptor and substrate levels found in the MNs. Despite this, it is interesting that the only gene found to be expressed in higher levels in MNs compared to the human brain is the receptor isoform *SV2C*, which is the isoform of SV2 with the highest affinity to BoNT/A [42]. The highest expression of *SV2C* was found for the protocol based on Du et al. [38], which had generated the highest proportion of MNs and might indicate a high sensitivity for BoNT/A.

The SiMa cells exhibited lower gene expression levels of receptors, which might indicate a lower sensitivity for BoNTs. Pathe-Neuschäfer-Rube et al. [56] showed a limited sensitivity of SiMa cells to BoNT/B compared to BoNT/A in a neurotransmitter release assay. In this study, the gene expression level of SYT1, which is the main receptor for BoNT/B in humans and primates, is expressed at lower levels in SiMa cells compared to the human brain and MNs (Figure 5), which might explain the lack of sensitivity [13].

The sensitivity of MNs generated with the protocol based on Du et al. [38] and SiMa cells to BoNT/A1 was analyzed via Western blot (Figure 6) by quantifying the percentage of cleaved SNAP25. It was found that the MNs have approximately 300 times lower IC_50_. The IC_50_ of the SiMa cells differs by a factor of 2-3 compared to similar studies that analyze SNAP25 cleavage by BoNT/A1 [57,58]. A recent study by Pellett et al. [24] analyzed SNAP25 cleavage in a population consisting of 87% MNs from a commercial source and found an IC_50_ of approximately 0.12 MLD/mL. The difference to the IC_50_ of approximately 0.97 MLD/mL found in this study for MNs differentiated with the protocol based on Du et al. [38] could be based on the lower proportion of MNs or experimental factors. Sensitivity of MNs could be further improved by using detection methods that quantify further steps of BoNT poisoning, such as neurotransmitter release. The higher sensitivity can, in part, be explained by the dominant negative effect of cleaved substrates on neurotransmitter exocytosis [59]. In the case of BoNT/A, one study reports that only 35% of SNAP25 needs to be cleaved to induce muscle paralysis [60]. Keller and Neale [61] analyzed spinal cord cell cultures and found a ten-fold lower IC_50_ for neurotransmitter release than for SNAP25 cleavage by BoNT/A [61]. This might explain the higher IC_50_ of approximately 279 MLD/mL found for SNAP25 cleavage in SiMa cells in this study, compared to the IC_50_ of approximately 71 MLD/mL for neurotransmitter release inhibition found in SiMa cells by Pathe-Neuschäfer-Rube et al. [26]. We, therefore, propose using human MNs as a highly sensitive and physiologically relevant model. The use of human MNs in combination with a neurotransmitter release assays might increase the sensitivity. The differentiation protocol based on Du et al. [38] has a good yield of MNs exhibiting expression of all major proteins required for detection of all BoNT serotypes at the gene and protein level and high expression of relevant receptor isoforms SV2C and SYT1. The sensitivity for BoNT/A1 was shown to be closely related to the mouse lethality assay.

## 4. Materials and Methods

### 4.1. Cell Culture

The human iPSC line IMR90-04 (IMR90) was originally purchased from WiCell and used for all differentiations [41]. Routine cultivation of IMR90 was conducted in StemMACS^™^ iPS-Brew XF medium (Miltenyi, Bergisch Gladbach, Germany) on plates coated with Corning^®^ Matrigel^®^ basement membrane preparation (high-concentrated, growth factor reduced, #354263, Corning, New York, USA). Medium was changed every other day and IMR90 were passaged with 0.02% Ethylenediaminetetraacetic acid (EDTA) in phosphate buffered saline without Mg^2+^ and Ca^2+^ (PBS). Additionally, 10 µM Rho-Kinase inhibitor Y-27632 (TargetMol, Boston, MA, USA) was added to the passage medium.

For differentiation to MNs, IMR90 were cultivated in neural medium, which consists of equal volumes of DMEM/F12 (Thermo Fisher Scientific, Waltham, MA, USA) and MACS^®^ Neuro medium (Miltenyi) as well as 0.5x N-2 Supplement (Thermo Fisher Scientific), 1x MACS^®^ NeuroBrew^®^-21 (Miltenyi), 1x L-glutamine (Biochrom, Berlin, Germany), and 1x penicillin/streptomycin (P/S, Sigma-Aldrich, Taufkirchen, Germany). All cells were cultivated at 37 °C, 5% CO_2_, 95% relative humidity, and checked for *Mycoplasma* contamination monthly. Additional information on cell culture supplements can be found in Table A1.

### 4.2. Differentiation by Du et al.

The protocol published by Du et al. [38] was applied with minor changes (Figure 1). Undifferentiated IMR90 were detached with 0.02% EDTA in PBS and seeded at 5∙10^4^ cells per mL in StemMACS^™^ iPS-Brew XF medium with 10 µM Y-27632 in 6-well plates coated with Matrigel. On the next day, the medium was exchanged with neural medium containing 3 µM CHIR99021 (CHIR, Axon Medchem, Groningen, Netherlands), 2 µM Dorsomorphin homolog 1 (DMH1) (Bertin Pharma, Montigny le Bretonneux, France), and 2 µM SB431542 (SB, Stemcell, Cologne, Germany). Furthermore, 100 µM of ascorbic acid (AA, Sigma) was added during the whole differentiation. The medium was changed every other day. On day 6, NESTIN-positive and SOX1-positive neural progenitor cells were split 1:6 with EDTA on Matrigel coated plates. Furthermore, 0.1 µM of retinoic acid (RA, Sigma) and 0.5 µM of Purmorphamine (PMA, Stemcell) was used in addition to CHIR, DMH1, and SB, while CHIR concentration was reduced to 1 µM. After 6 days in this medium, OLIG2-positive motor neuron progenitors (pMNs) could be detected. These can either be expanded for a few passages or differentiated further. For expansion, pMNs were detached and split regularly with EDTA and cultured in neural medium containing 3 µM CHIR, 2 µM DMH1, 2 µM SB, 0.1 µM RA, 0.5 µM PMA, and 0.5 mM valproic acid (Sigma) on Matrigel-coated plates. For maturation into MNs, pMNs were detached with 1 mg/mL collagenase IV (Thermo Fisher Scientific) and transferred to low-attachment plates (Corning) in neural medium with 0.5 µM RA and 0.1 µM PMA. The resulting neurospheres were kept in suspension for 6 days, dissociated into single cells with Accutase (Sigma-Aldrich), and seeded at 2∙10^5^ cells per mL in Matrigel-coated plates. After maturation for 10 days in neural medium containing 0.5 µM RA, 0.1 µM PMA, 0.1 µM compound E (CE, Bertin Pharma), 2 ng/mL of glia-derived neurotrophic factor (GDNF), brain-derived neurotrophic factor (BDNF), and ciliary neurotrophic factor (CNTF) (all from Peprotech, Hamburg, Germany) cells matured into ISLET1- and CHAT-positive MNs.

### 4.3. Differentiation by Maury et al.

The differentiation based on the protocol by Maury et al. [39] was conducted as following (Figure 1): IMR90 were detached with EDTA-solution and transferred at 1∙10^5^ cells/mL as small aggregates into low-attachment plates in neural medium containing 0.5 µM AA, 3 µM CHIR, 2µM DMH1, 2 µM SB, and 5 µM Y-27632 and cultivated in suspension. On day 2, Y-27632 was withdrawn and 0.1 µM RA and 0.5 µM smoothened agonist (SAG, TargetMol) were supplemented. From day 4 to day 9, only AA, RA, and SAG were added to the medium. On day 9, the OLIG2-positive neurospheres were dissociated with Accutase and seeded at 2-3∙10^5^ cells per mL neural medium in Matrigel-coated plates. In addition, 10 µM tert-Butyl (2S)-2-[[(2S)-2-[[2-(3,5-difluorophenyl)acetyl]amino]propanoyl]-amino]-2-phenylacetate (DAPT, Cayman Chemicals) was added from day 9 to day 14. From day 11, neurotrophic factors GDNF, BDNF, and CNTF (5 ng/mL) and 1 µg/mL dbcAMP (Sigma) were added. The resulting ISLET1-positive and CHAT-positive mature MNs were analyzed on day 32.

### 4.4. Differentiation by Kroehne et al. 

The protocol by Kroehne et al. [40] is based on the generation of neural progenitor cells (NPCs), which was originally published by Reinhardt et al. [44]. These NPCs can be expanded without limitation and then differentiated to MNs (Figure 1). For the generation of NPCs, IMR90 were detached from Matrigel-coated plates with ETDA and cultivated on a feeder layer of mouse embryonic fibroblasts (MEFs, Cell Biolabs Inc., Heidelberg, Germany), which were mitotically inactivated with Mitomycin C (Sigma). hES medium was used, which consisted of DMEM/F12 with 20% knock-out serum replacement (Thermo Fisher Scientific), 1× P/S, 1 × L-glutamine, 1x non-essential amino acids (Thermo Fisher Scientific), 100 µM ß-mercaptoethanol (Thermo Fisher Scientific), and 5 ng/mL basic fibroblast growth factor (bFGF, Thermo Fisher Scientific). Medium was changed every day during the co-culture. IMR90 formed colonies on the feeder layer, which were detached with 1 mg/mL Collagenase IV and disaggregated mechanically for passaging. After passaging, 10 µM Y-27632 was added for a day. After one week, embryoid bodies were generated by cutting the iPSC colonies into pieces and detaching the resulting cell aggregates with collagenase. The embryoid bodies were cultivated in low-attachment plates and the medium was changed every other day. hES medium with 1 µM Dorsomorphin (DM, Abcam, Berlin, Germany), 3 µM CHIR, 0.5 µM PMA, and 10 µM SB was used for the first 2 days. Then neural medium was used with the same supplements. After day 4, neural medium containing 3 µM CHIR, 0.5 µM PMA, and 150 µM AA was used. On day 6, about 50 embryoid bodies were transferred to one well of a 12-well plate coated with Matrigel and mechanically dissociated with a pipette. The resulting NPCs were split about once every week with Accutase and cultivated with this medium for 3 passages. From passage 4 on, 0.5 µM SAG was used instead of PMA. NPCs were passaged for at least 13 times before they were used for differentiation into MNs. For MN differentiation, 2∙10^5^ NPCs/mL were seeded in Matrigel-coated plates in neural medium supplemented with 200 µM AA, 0.5 µM SAG, 1 µM RA, 1 ng/mL GDNF, and 2 ng/mL BDNF. After 6 days, OLIG2-positive pMNs were re-plated on Matrigel and maturated in neural medium containing 200 µM AA, 2 ng/mL GDNF and BDNF, 1 ng/mL TGFß3 (Sigma), 200 µM dbcAMP, and 10 µM DAPT until day 21.

### 4.5. Cultivation and Differentiation of SiMa Cells

The neuroblastoma cell line SiMa (SiMa-hPOMC1-26GLuc) was kindly provided by G. Püschel and was originally obtained from the DSMZ (ACC 164) [62]. For neurotransmitter release quantification, a luciferase reporter was integrated, which is not expected to affect this study [26]. SiMa cells were cultivated in RPMI1640 (Biochrom) with 10% inactivated FCS (Biochrom), 1% P/S, 1% L-glutamine, and passaged once per week with trypsin/EDTA (Biochrom). For partial differentiation into a more neuronal phenotype, SiMa were seeded on poly-L-lysine (Sigma) coated 6-well plates and cultivated in differentiation medium (RPMI1640 with 2% MACS^®^ NeuroBrew^®^-21, 1% N-2 Supplement, 1mM non-essential amino acids, 1% P/S, 1% L-glutamine). SiMas were differentiated for four days prior to expression level analysis and for two days before BoNT/A1 treatment.

### 4.6. Quantitative RT-qPCR

At the specified time points, cultured cells were collected and stored at −80 °C until total RNA was extracted from with TRIzol (Thermo Fisher Scientific) and isolated on Nucleospin RNA columns (Macherey-Nagel, Düren, Germany), according to the manufacturer’s recommendations. DNA was removed by incubating with RQ1 RNase-free DNase (Promega Corporation, Madison, WI, USA) in the presence of RiboLock RNAse inhibitor (Thermo Fisher Scientific). For reverse transcription to cDNA, RevertAid reverse transcriptase (Thermo Fisher Scientific) was used with dNTPs Roti^®^-Mix PCR 3 (Roth, Karlsruhe, Germany) and random hexamer primers (Thermo Fisher Scientific). Gene expression levels of genes of interest were analyzed in triplicate with RT-qPCR with SYBR green (Thermo Fisher Scientific) in an Mx3000P qPCR cycler (Agilent Technologies, Santa Clara, CA, USA). Furthermore, 20 ng of cDNA was amplified with 0.25 µM of the respective primers (Table A2) and DreamTaq™ Hot Start DNA polymerase (Thermo Fisher Scientific) in 40 cycles of 15 s at 95 °C/60 s at 60 °C. No template control was used with each amplification. The quantification cycle (C_q_) was determined with the MxPro-Mx3000P software and relative gene expression levels were calculated with the 2^-∆C^_q_ method [63] and normalized to the geometric mean of the reference genes cyclophilin A (*PPIA*) and ribosomal protein S23 (*RPS23*). Both reference genes have been shown to be stably expressed using the geNorm algorithm implemented in qBase+ software, version 3.0 (Biogazelle, Zwijnaarde, Belgium, www.qbaseplus.com) [64].

### 4.7. Immunocytochemistry

At the specified time points, cells grown on Matrigel-coated glass coverslips were prefixed by adding an equal volume of formaldehyde solution (4% in PBS) to the medium. After 10 min, fresh formaldehyde solution was added and incubated for another 10 min. Coverslips were washed twice with PBS and stored in PBS at 4° C. Cells were permeabilized with 0.25% triton-X in PBS for 10 min, nonspecific binding reduced by adding 5% serum and 1% bovine serum albumin (BSA) in PBS with 0.01% Tween-20 (PBST) for 1h. Primary antibodies (Table A3) were diluted in 1% BSA in PBST and incubated overnight at 4° C. After washing three times with PBS, secondary antibodies (Table A4) were added in 1% BSA in PBST for 1 h at room temperature. After washing three times with PBS, coverslips were mounted with ProLong™ Gold Antifade Mountant with DAPI (Thermo Fisher Scientific) and imaged with an inverse fluorescence microscope Axiovert 200M (Zeiss). For quantification of MNs, ILSET1-positive cells were counted by a person blind to the experiment from an average of 200-400 cells in random fields from at least three independent differentiations with the aid of the CellCounter plug-in for ImageJ (Version 1.51q for Windows, ImageJ, U. S. National Institutes of Health, Bethesda, MD, USA, https://imagej.nih.gov/ij). Statistics were calculated with GraphPadPrism version 8.3.0 for Windows (GraphPad Software, San Diego, CA, USA, www.graphpad.com).

### 4.8. Analysis of SNAP25 Cleavage

Immature MNs generated by the protocol based on Du et al. [38] were frozen on day 18 of the differentiation and thawed when needed. The 150 kDa protein of purified BoNT/A1 was purchased from Miprolab (Göttingen, Germany; #3101-0010). The potency was determined by the manufacturer (0.28 minimum lethal doses per pg). After 10 days in culture, MNs were treated with BoNT/A1 diluted in neural medium for 48 h. SiMa cells were differentiated for two days and treated with BoNT/A1 in differentiation medium. After the treatment, all cells were washed two times with PBS and lysed with RIPA buffer and ultrasonication. In addition, 10 µg of MN lysate or 25 µg of SiMa lysate was separated with pre-casted Any kD™ Mini-PROTEAN^®^ TGX™ Gels (Bio-Rad, Feldkirchen, Germany), transferred onto a nitrocellulose membrane (GE Healthcare, Freiburg, Germany), and then incubated over night with anti-SNAP25 primary antibody (Table A3) diluted to 1 µg/mL in blocking buffer (5% milk powder, 0.2% Tween-20 in Tris-buffered saline). After washing, the horseradish peroxidase (HRP) coupled secondary antibody (Table A4) diluted in blocking buffer was added for 1 h at room temperature. HRP-coupled anti-β-actin antibody (Table A3) in blocking buffer was used to detect Actin, which was used as a loading control. β-actin and SNAP25 bands were quantified by dosimetry. SNAP25-cleavage was modelled by nonlinear regression (four parameters, variable slope) and analyzed using GraphPad Prism version 8.3.0 for Windows (GraphPad Software, San Diego, CA, USA, www.graphpad.com).

## Figures and Tables

**Table 1:** Botulinum neurotoxins (BoNTs), the respective ganglioside, and protein receptors as well as substrates that are relevant for inhibition of neurotransmission. The affinity to the isoforms of the targeted molecules is ordered by decreasing specificity. (SNAP25: Synaptosomal-associated protein 25, SV2: Synaptic Vesicle Protein, VAMP: Vesicle-associated Membrane Protein, STX: Syntaxin, SYT: Synaptotagmin). Data was obtained from References [6,9,10,11,12,13].

BoNT	Ganglioside Receptor [12]	Protein Receptor [12]	Substrate [6]	Cleavage Site [6]
**A**	GT1b > GD1a = GD1b > GM1	SV2C>SV2A>SV2B	SNAP25	Q197–198R
**B**	GT1b > GD1a > GD1b	SYT1 >SYT2 *	VAMP1 VAMP2	Q78–79F Q76–77F
**C**	GD1b > GT1b > GD1a > GM1a	Not determined	SNAP25 STX1A STX1B	R198–199A K253–254A K252–253A
**DC**	GM1a > GD1a > GD1b = GT1b>	SYT2 >SYT1	VAMP1 VAMP2	K61–62L K59–60L
**D**	GD2 > GT1b = GD1b	SV2B>SV2C>SV2A	VAMP1 VAMP2	K61–62L K59–60L
**E**	GD1a/GQ1b/GT1b >> GM1	SV2A>SV2B	SNAP25	R180–181I
**F**	GT1b = GD1a >> GM3 >> GD1b/GM1	SV2A>SV2C>SV2B	VAMP1 VAMP2	Q60–61K Q58–59K
**G**	GT1b = GD1a > GD1b > GM3 > GM1	SYT1, SYT2	VAMP1 VAMP2	A83–84A A81–82A
**H/FA**	Not determined	SV2	VAMP1 VAMP2	L56–57E L54–55E

* in primates and humans, due to a mutation in the *SYT2* gene [13].

**Figure A1:**
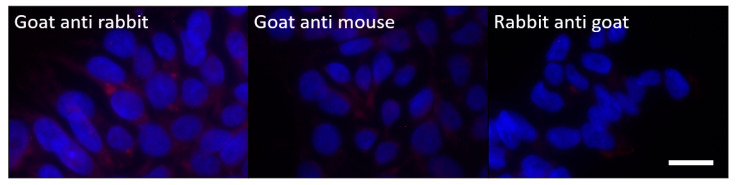
Negative controls for the antibodies used in Figure 2 and Figure 4. Undifferentiated IMR90 were used and the primary antibody was omitted. Scale bar = 10 µm.

## Appendix A

**Table A1:** Cell culture supplements.

Supplement	Supplier	Stock Solution
Y-27632 (Y)	Bertin Pharma #T1725	10 mM in H_2_O
bFGF	Gibco #13256029	5 µg/mL in 10 mM Tris, pH 7.6 with 0.1% BSA
Dorsomorphin (DM)	abcam #ab120843	10 mM in DMSO
CHIR99021 (CHIR)	Axon MedChem #1386	6 mM in DMSO
Valproic acid (VPA)	Sigma #P6273	10 mM in DMEM/F12
SB431542 (SB)	Stemcell #72232	40 mM in DMSO
L-Ascorbic acid (AA)	Sigma #A4544	150 mM in H_2_O
TGFß3	Sigma #SRP3171	10 µg/mL in 5 mM citric acid with 0.1% BSA
DMH1	Bertin Pharma, #16679	10 mM in DMF
Purmorphamine (PMA)	Stemcell #72202	10 mM in DMSO
Compound E (CE)	Bertin Pharma, #15579	10 mM in DMSO
GDNF	Peprotech, #450-10	10 µg/mL in 0.1% BSA
BDNF	Peprotech, #450-02	10 µg/mL in 0.1% BSA
CNTF	Peprotech, #450-13	10 µg/mL in 0.1% BSA
SAG	TargetMol #T1779	2.5 mM in DMSO
DAPT	Cayman Chemicals #Cay13197-5	10 mM in DMSO
dbcAMP (AMP)	Sigma #D0627	100 mM in H_2_O
Retinoic acid (RA)	Sigma #R2625	10 mM in DMSO, protect from light

**Table A2:** Primers used for gene expression level quantification.

Gene	Forward Primer (5′-3′)	Reverse primer (5′-3′)
***PPIA*** NM_001300981.2	GCCAAGACTGAGTGGTTGGAT	GGCCTCCACAATATTCATGCC
***RPS23*** NM_001025.5	ACAGGATGGGCAAGTGTCGT	CACTTCTGGTCTCGTCGGTG
***SNAP25*** NM_001322902.2	AGCCTGGGGCAATAATCAGG	GGCATCATTTGTTACCCTGCG
***STX1A*** NM_001165903.2	CAACCCCGATGAGAAGACGA	GGCGTTGTACTCCGACATGA
***STX1B*** NM_052874.5	GAAGGACCACCACCAACGAA	ATCTCTCCCTGGCTCTCTACG
***VAMP1*** NM_001297438.2	CAGTTCCGTCCACTTCAGCC	CTGGAGCAGACATTTTTCTGACA
***VAMP2*** NM_001330125.1	CCAAACCTCACCAGTAACAGGA	CTCATGATGTCCACCACCTCA
***SV2A*** NM_001278719.1	CCTCAGACAAGAGGACCACAG	GCCCTAGAGACCCCTTCACT
***SV2B*** NM_001167580.3	CCACCAACATGGGAAACTTGTG	GTGCTCGTAGAGGTCTGTGTT
***SV2C*** NM_001297716.2	TCGGGATTGGAGGAGCCATA	ATGCTGAAGCTCCACCCGTA
***SYT1*** NM_001135805.2	GGATGTGGGTGGCTTATCCG	CCACCTGCACTTTCTGGATTTG
***SYT2*** NM_001136504.1	CTTCAAGGTGCCATACCAGGA	CTCCACTCCTCAATGGGCTG
***ST3GAL2*** NM_006927.3	TGAGAGTGCCAAGAACCTGC	CTGGGGCGTAGGTGAATCG
***ST3GAL3*** NM_001270459.1	ATCTTCCCCCGGTTCTCCAA	CGAACTCCCGGATTCTAGCC

**Table A3:** Primary antibodies used in this study.

Antibody	Host and Type	Source	Dilution
OCT4	Mouse monoclonal	Santa Cruz #sc-5279	1:50
SOX2	Rabbit monoclonal	Cell Signaling #3579	1:200
OLIG2	Rabbit polyclonal	Merck/Millipore #AB9610	1:250
NKX6.1	Mouse monoclonal	DSHB #F55A10	5 µg/mL
TUJ1	Rabbit polyclonal	Sigma #T2200	1:100
TUJ1	Mouse monoclonal	Covance #MMS-435P	1:750
ISLET1	Rabbit polyclonal	Abcam #ab20670	1:125
CHAT	Goat polyclonal	Millipore #AB144P	1:50
SNAP25	Rabbit polyclonal	Abcam #41455	1:100
VAMP2	Rabbit monoclonal	Cell signaling #D601A	1:250
SV2 (SV2A/B/C)	Mouse monoclonal	DSHB #SV2	10 µg/mL
GT1b	Mouse monoclonal	DSHB #GT1b-1	10 µg/µL
GD1a	Mouse monoclonal	DSHB #GD1a-1	10 µg/mL
β-Actin (HRP-coupled)	Mouse monoclonal	Santa Cruz #sc-47778	1:4000

GT1b-1 and GD1a were deposited to the Developmental Studies Hybridoma Bank (DSHB) by Schnaar, R.L. SV2 was deposited to the DSHB by Buckley, K.M. NKX6.1 was deposited to the DSHB by Madsen, O.D.

**Table A4:** Secondary antibodies used in this study.

Antibody	Host and Type	Source	Dilution
Anti-Goat (Alexa Fluor 568)	Rabbit polyclonal	Invitrogen #A11079	1:1000
Anti-Mouse (Alexa Fluor 488)	Goat polyclonal	Invitrogen #A11001	1:1500
Anti-Rabbit (Alexa Fluor 568)	Goat polyclonal	Invitrogen #A11008	1:1000
Anti-Rabbit (HRP)	Goat polyclonal	Sigma #A0545	1:20,000
